# Coagulation temperature and smoking time determine product quality and shelf life of the acid‐heat coagulated Circassian cheese

**DOI:** 10.1002/fsn3.3552

**Published:** 2023-07-06

**Authors:** Hatice Sıçramaz, Ahmet Ayar

**Affiliations:** ^1^ Faculty of Engineering, Department of Food Engineering Sakarya University Sakarya Turkey

**Keywords:** acid‐heat coagulated cheese, cheese quality, cold‐smoking, microstructure, shelf life

## Abstract

This study examined the effects of process parameters on acid‐heat coagulated Circassian cheese. For this purpose, cheeses were produced at coagulation temperatures of 70°C and 90°C, and smoked for 0, 2.5, and 6 h in both summer and winter. Microbiological, textural, proteolytic, and sensorial changes were observed for 90 days at 30‐day intervals. According to the results, coagulation at 90°C instead of 70°C formed a firmer structure. Six‐hour smoking time instead of 2.5‐h provided higher dry matter, reduced proteolysis rates, and extended the microbial shelf life. In addition, higher (6 h) smoking decelerated sourness while resulting in intense smoke flavor and higher lipolytic activity. In conclusion, seasonal changes in milk and applied the process conditions revealed significant differences in the quality parameters and shelf life of acid‐heat coagulated Circassian cheese.

## Graphical abstract

Process parameters of acid‐heat coagulated cheeses were investigated. In this context, the effects of coagulation temperature, smoking time, seasonal changes, and storage time were evaluated in terms of textural, microstructural, and ripening properties of cheese. This article will contribute to the industry in determining the process parameters of acid‐heat coagulated cheeses and will provide solutions to fill the gap in the literature.

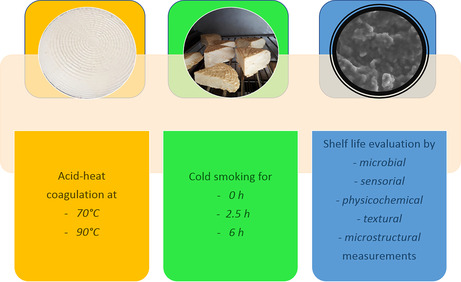

## 1 INTRODUCTION

Circassian cheese is a traditional acid‐heat coagulated cheese of the Adyghe people who lived in the North Caucasus region of Southern Russia and migrated to Turkey, Jordan, Syria, Saudi Arabia, Germany, and the United States after 1858 (Bukvić, 2016) and therefore it is also called Adyghe cheese. Although there is no information about the production capacity of the cheese in the world, it was stated that there is an annual market demand of 12,000 tons in the Republic of Adygea (Anonymous, 2018). It is stated that in the traditional production method of Circassian cheese, whole milk at nearly boiling temperature is precipitated with an acidic juice of sour milk, very sour whey, and vinegar. Then the curd is placed in a strainer and salted (Jaimoukha, 2004). There is a Russian GOST standard for Circassian cheese named Adyghe cheese which includes provisions on general food hygiene, additives, pesticide residues, and the general composition of the final product (ISC, 2014). However, there is still diversity and disagreement in the cheese production steps (Pryanichnikova, 2020), and the process details are not the subject of the GOST standard. Circassian cheese does not yet have a PGI, and the effects of different applications on the product have not yet been scientifically investigated.

Although acid‐heat coagulated cheeses are subject to minimal processing differences compared to chymosin‐coagulated cheeses, minor differences in their production affect compositional, textural, sensory, and other main characteristics of the cheese. Production of acid‐heat coagulated cheese varieties such as mascarpone, ricotta, paneer, and queso blanco typically involves heating milk to 80–90°C, acidifying to a pH in the range of 5.4–6.0, and recovering curds. The quality of these fresh cheeses is affected by many parameters, including the gel structure and the treatments applied to the curd (Fox et al., 2017). There are many studies examining the effects of changes in the production process on the properties of milk gel (Brighenti et al., 2018; Lucey, 2002; Vasbinder et al., 2001) but studies on the effects on the final product are limited. The effects of coagulation temperature differences in the yield and texture of acid‐heat coagulated cheeses were investigated by Hydamaka et al. (2001), but mainly, studies in the literature focus on rennet‐coagulated cheeses instead of acid‐heat coagulated ones. In addition, there are application differences in the coagulation temperature of acid‐heat coagulated Circassian cheese (Gul et al., 2019; Jaimoukha, 2004; Parlak & Güzeler, 2017; Sıçramaz et al., 2017). Although it is generally consumed as smoked, the method and time of smoking do not have a standard practice.

The smoking process is the oldest known food preservation method, and today it is mostly applied for the flavor preferences of consumers (Ogbadu, 2014). The phenolic compounds in the smoke both extend the shelf life with their antioxidant properties and add different flavors to the final product (Ahmad, 2003). Smoking time is one of the important factors and a very critical step that affects the phenolic compounds in the product. However, the effect of different smoking times on the Circassian cheese has not yet been determined. In previous studies, the volatile compounds of the Circassian cheeses obtained from the market have been examined (Gul et al., 2015; Guneser & Yuceer, 2011). Some studies have been carried out to improve the traditional cheese production process, but no research on smoking time has been done in these studies (Gul et al., 2019; Parlak & Güzeler, 2017; Sıçramaz et al., 2017).

Although the effect of coagulation temperature on some properties of acid‐heat coagulated cheeses has been investigated previously, adequate shelf life assessments have not been made and the contribution of smoking time has not been demonstrated. In addition, there is no standard production method for acid‐heat coagulated Circassian cheese. This study aims to investigate the effect of coagulation temperature and smoking time on the quality parameters and shelf life of acid‐heat coagulated cheeses, considering that it will also contribute to the standardization of the production method of Circassian cheese.

## 2 MATERIALS AND METHODS

### 2.1 Preparation of sour whey to be used as coagulant

Approximately 77 kg of acid whey (7.3% nonfat dry matter [NFDM], pH 4.8) was obtained seasonally from feta cheese production in Güneşoğlu dairy plant (Sakarya, Turkey). The acid whey was further cold stored until the pH decreased to 3.2. Then it was pasteurized at 90°C for 15 min to kill vegetative cells before use. The product was called “sour whey.”

### 2.2 Supply of milk

Holstein Friesian cow's milk was obtained from a farm in Sakarya. In order to observe seasonal differences, productions were carried out in triplicate with both summer and winter milk in 2018. The average compositions of raw milk samples obtained in the first 3 weeks of August and February are given in Table 1.

**TABLE 1:** The chemical compositions of raw milk samples used in cheesemaking.

	Summer (August)	Winter (February)
Fat (%)	2.9 ± 0.05 B	3.9 ± 0.01 A
Protein (%)	3.3 ± 0.04 B	3.5 ± 0.01 A
NFDM (%)	8.7 ± 0.09 B	9.7 ± 0.03 A
pH (at 10°C)	6.6 ± 0.02 A	6.6 ± 0.01 A

*Note*: Capital letters in the same line represent seasonal variations of the specified characteristics of the sample (*p* < .05).

### 2.3 Cheesemaking

The cheeses were produced in triplicate with milk from independent batches in both summer (August) and winter (February). Raw milk was pasteurized in 200 kg batches in a double‐jacketed tank at 90°C for 5 s. One group of cheeses was coagulated at 90°C and the other group was immediately cooled after pasteurization and coagulated at 70°C. Immediately after reaching the coagulation temperature, 12.8% sour whey and 0.054% lactic acid (food grade, Smart Chemistry) were used to decrease the pH from 6.7 to 5.2–5.0. After acidification, milk was aggregated and clotted for 5 min with gentle stirring, and then the filtration process was started. Plastic sieves with one 1 kg of cheese capacity were used for whey draining and molding. The obtained curd was dry salted at a rate of 1% (w/w). Cheeses were kept overnight in a cold room at 4°C for further draining.

After drainage, each 1 kg of cheese was divided into six equal parts. Fresh cheese group (F) was packaged under vacuum using polyamide/polyethylene (PA/PE) bags. The remaining cheese was divided into two groups to be smoked at different times. A temperature‐controlled oven combined with a cold‐smoke generator supplied by Gıdamaksan (Sakarya, Turkey) was used in the smoking process. Cheeses were smoked at 25°C by a cold‐smoking method by burning oak dust. The two smoked cheese groups were smoked for 2.5 and 6 h, respectively, and the low‐smoked (2.5 h) group was coded as “LS” and the high‐smoked (6 h) group as “HS.” The experimental design used in the study is given in Table 2. All products were vacuum packed and stored at 4°C until the day of analysis. The general compositions of the cheeses were determined on the 15th day of storage, and microbiological, proteolytic, lipolytic, textural, and sensory properties during the shelf life were investigated on the 1st, 30th, 60th, and 90th days with three replications. Sampling for the analysis was done by grating the cheese to include the smoked outer skin of the cheese. Microstructures of cheeses were visualized on days 1 and 90.

**TABLE 2:** Cheese groups and process details.

Product code	Coagulation temperature (°C)	Smoking time (h)
70‐F	70	–
70‐LS	70	2.5
70‐HS	70	6
90‐F	90	–
90‐LS	90	2.5
90‐HS	90	6

*Note*: Products were coded according to the coagulation temperature and the smoking time – 70 and 90: *coagulation temperatures* (°C); F: *fresh*, LS: *2.5 h smoked*, HS: *6 h smoked*.

### 2.4 Compositional analysis

The dry matter (DM; IDF, 2004), fat (ISO, 2008), protein (IDF, 2001), and salt (ISO, 2006) analyses were carried out according to the international standard methods. The pH of the cheeses was analyzed using pH meter (Hanna Instruments, Germany) after mixing 10 g of grated cheese sample with 10 mL of distilled water with the help of an IKA T18 (IKA‐Werke GmbH Co., Germany) homogenizer. The samples for titration acidity analysis were prepared with the same method, and the acidity in terms of lactic acid (%LA) was calculated by titration against sodium hydroxide (NaOH).

### 2.5 Microbiological analysis

Serial dilutions of cheeses were prepared in Ringer's solution for microbial count inoculations. Plate count agar (PCA) was used for total mesophilic aerobic bacteria (TMAB), oxytetracycline glucose yeast extract agar (OGYEA) was used for total yeasts and molds, and violet red bile agar (VRBA) was used for coliform bacteria. All the media were supplied by Merck (Germany). Colonies formed in the Petri dishes were counted after 48 h of incubation at 35°C in PCA, 5–7 days at 25–30°C in OGYEA, and 24 h at 37°C in VRBA (Bridson, 1998).

### 2.6 Assessment of proteolysis and lipolysis

The amount of water‐soluble nitrogen (WSN) is an indicator for determining the level of proteolysis. In the WSN content analysis, the water‐soluble nitrogenous substances were passed into the water with the method of Kuchroo and Fox (1982), and the total amount of nitrogenous compounds was determined by the Kjeldahl nitrogen analysis (IDF, 2001). The ratio of WSN value to total nitrogen (TN) was calculated as the degree of ripening (RD) according to the formula: %RD = %WSN × 100/%TN.

Lipolysis level in cheeses during shelf life was measured according to the total free fatty acid (TFFA) determination method applied by Nuñez et al. (1986), with some modifications. In the method, 10 g of ground cheese was mixed thoroughly with 6 g of anhydrous sodium sulfate (Na_2_SO_4_) (Merck, Germany) in a mortar. The mixture was transferred to a flask with 60 mL diethyl ether (Merck, Germany). The homogenate was kept for 1 h, with shaking vigorously for 1 min at 15‐min intervals. Then, the mixture was filtered through Whatman no. 40 paper by washing three times with 20 mL portions of diethyl ether. The solvent of the permeate was separated by vacuum evaporation (Buchi rotavapor R‐215). The collected free fatty acids were titrated by 0.05 N potassium hydroxide (KOH) prepared in diethyl ether:ethanol (1:1, v:v) solution using phenolphthalein as indicator. Titrant volumes (mL) for sample and blank without sample were abbreviated as *Vsample* and *Vblank*, respectively. The TFFA results were calculated according to the formula (1) and were expressed as “g oleic acid per 100 g total fat.”

(1) $$\;\text{TFFA}=\frac{\left(\text{Vsample}-\text{Vblank}\right)\;x\;\left(282\frac{g}{\mathrm{mol}}\text{oleic acid}\right)x\;0.05\;N\;\mathrm{KOH}}{10\;g\;\text{sample}\;x\;100\;g\;\text{total}\;\mathrm{fat}}$$

### 2.7 Textural analysis

Cheese cut into triangles with a thickness of 8 cm was tested using Brookfield CT3‐4500 g texture analyzer (AMETEK Brookfield, USA). The hardness of cheeses was determined by TA 15/1000 45° conical probe at 10°C with a test speed of 1 mm/sec and a trigger load of 4 g at a penetration depth of 20 mm by compression test, and the results were given in grams.

### 2.8 Sensory analysis

Sensory characteristics were analyzed by eight panelists aged between 24 and 50, consisting of trained male and female staff of Sakarya University Department of Food Engineering. In the sensory analysis, the cheeses were evaluated in terms of color, hardness, elasticity, sourness, saltiness, smoked flavor intensity, and general acceptance. The sensory analysis method used in the panel was a hybrid method that includes a hedonic test question to assess liking, as well as a quality rating test with a score table of 1–9 points scale (Lawless & Heymann, 2010). In the evaluation, the scoring was designed as follows: for color – 1: *white*, 9: *yellow*; for hardness, elasticity, sourness, saltiness, and smoked flavor intensity – 1: *none*, 9: *extremely high*; for general acceptability – 1: *dislike*, 9: *like very much*.

### 2.9 Measurement of microstructural characteristics

The microstructures of the cheeses were examined in the FEI Quanta FEG 250 scanning electron microscope in ESEM mode using a gaseous secondary electron detector (GSED) according to the method of Noronha et al. (2008) with some modifications. Five mm cubic pieces of cheese were sampled and mounted on a Peltier cooling stage set at 5°C. The samples were examined at a working distance of 5.8–6.5 mm and an accelerating voltage of 30 kV, using the wet mode at a water vapor pressure of 7.25 Torr. Images were obtained on the 1st and 90th days of storage at 1000× magnification.

### 2.10 Statistical analysis

The homogeneity of the data was analyzed using SPSS 20.0 (IBM, USA) software, according to Tabachnick and Fidell (2012). In determining the seasonal differences between the cheeses, *T* test was applied for comparison of dependent variables at a significance level of *p* < .05. In order to examine all other differences between the cheeses, an ANOVA test was performed using Tukey's HSD multiple comparison method at a significance level of *p* < .01 using SPSS 20.0. Principal component analysis was performed using Minitab 16 (Minitab Inc.) to evaluate sensory scores and ripening values. Summer and winter products were produced with three independent production repetitions, and smoking processes of these repetitions were also carried out in different batches. Analyzes were performed in at least three replicates for each batch.

## 3 RESULTS AND DISCUSSION

### 3.1 Cheese composition

The average composition of the cheese on day 15 is shown in Table 3. Seasonal changes caused a difference in the DM of cheeses. It has been observed that cheeses produced with summer milk have significantly lower DM than cheeses produced with winter milk. This was an expected result because the fat and protein values of summer milk were 2.9% and 3.30%, respectively, while those of winter milk were 3.9% and 3.50%, respectively. Similarly, Larsen et al. (2014) obtained the lowest fat rate in milk in summer and the highest in winter months. The protein values have been reported in a wide range of 2.53%–3.53% for summer and 2.66%–3.56% for winter milk, in the literature (Büyükoğlu et al., 2017; Lin et al., 2017). However, when examined in DM, protein and fat ratios of cheeses were not affected by seasonal changes. Contrary to our results, Jaeggi et al. (2005) reported that the difference in fat in February and August cheeses was significantly different in both the total and dry matter of the cheese. Another parameter investigated in this study was the effect of smoking time, and it was observed that 2.5 and 6 h smoking did not affect protein and fat ratios in DM. Low‐smoked (2.5 h) cheese group was similar to fresh cheeses, while cheeses smoked for 6 h revealed higher DM, probably due to drying during smoking. Another observation within the scope of the study was the effect of coagulation temperature, and the results were similar to the study of Hydamaka et al. (2001). While the DM of cheeses coagulated at 90°C was higher than cheeses coagulated at 70°C, protein and fat ratios in DM were not affected by coagulation temperature. According to the literature, an increase in temperature increases the probability of the protein remaining in the cheese curd (Law et al., 1994). However, since the products coagulated at 70°C were also previously heated to 90°C, the protein ratios in DM were not affected by the coagulation temperature.

**TABLE 3:** The average cheese compositions.

	Summer						Winter					
	DM	Fat	Protein	Salt	Fat in DM	Protein in DM	DM	Fat	Protein	Salt	Fat in DM	Protein in DM
70‐F	41.6 ± 0.11	43.6 ± 0.08	23.3 ± 0.29	24.3 ± 0.29	17.2 ± 0.10	18.1 ± 0.09	43.6 ± 0.08	24.3 ± 0.29	18.1 ± 0.09	1.23 ± 0.06	55.8 ± 0.56	41.4 ± 0.14
	B c	A c	B b	A b	B d	A d	A c	A b	A d	a	A a	A b
70‐LS	42.0 ± 0.21	43.9 ± 0.53	23.3 ± 0.29	24.3 ± 0.29	17.6 ± 0.25	18.3 ± 0.10	43.9 ± 0.53	24.3 ± 0.29	18.3 ± 0.10	1.23 ± 0.28	55.4 ± 0.89	41.6 ± 0.64
	B c	A c	B a	A b	B cd	A d	A c	A b	A d	a	A a	A b
70‐HS	43.8 ± 0.64	46.0 ± 0.30	23.8 ± 0.29	25.0 ± 0.50	19.0 ± 0.25	19.8 ± 0.10	46.0 ± 0.30	25.0 ± 0.50	19.8 ± 0.10	1.13 ± 0.02	54.3 ± 0.77	43.0 ± 1.16
	B b	A b	B ab	A ab	B b	A b	A b	A ab	A b	a	A a	A a
90‐F	43.6 ± 0.25	45.9 ± 0.45	24.3 ± 0.29	25.7 ± 0.29	18.3 ± 0.09	18.8 ± 0.11	45.9 ± 0.45	25.7 ± 0.29	18.8 ± 0.11	1.11 ± 0.03	56.0 ± 0.23	41.1 ± 0.26
	B b	A b	B ab	A a	B bc	A c	A b	A a	A c	a	A a	A b
90‐LS	43.7 ± 0.51	46.7 ± 0.04	23.8 ± 0.29	25.3 ± 0.29	19.0 ± 0.19	20.1 ± 0.09	46.7 ± 0.04	25.3 ± 0.29	20.1 ± 0.09	1.19 ± 0.03	54.2 ± 0.64	43.1 ± 0.81
	B b	A b	B ab	A ab	B b	A b	A b	A ab	A b	a	A a	A a
90‐HS	45.8 ± 0.45	48.6 ± 0.38	24.7 ± 0.29	26.2 ± 0.29	19.9 ± 0.23	21.0 ± 0.30	48.6 ± 0.38	26.2 ± 0.29	21.0 ± 0.30	1.20 ± 0.03	53.8 ± 1.02	43.3 ± 0.08
	B a	A a	B a	A a	B a	A a	A a	A a	A a	a	A a	A a

*Note*: 70 and 90: *coagulation temperatures* (°C), ‐F: *fresh*, ‐LS: *2.5 h smoked*, ‐HS: *6 h smoked*, DM: *dry matter*. All measurements were given in percent (%). Lowercase letters in the same column indicate a difference in the specified property between samples (*p* < .01). Capital letters in the same line represent seasonal variations of the specified characteristics of the sample. (*p* < .05).

### 3.2 Microbiological properties

Coliform group bacteria were not found in any of the cheeses during their shelf life. The TMAB and yeast and mold counts are given in Table 4. Microbial quality was not affected by coagulation temperature. This was an expected result, because both (90°C and 70°C coagulated) cheese groups were pasteurized at 90°C for 15 s. Six‐hour smoking had a positive contribution to the extension of the microbial shelf life according to the TMAB and yeast and mold counts during the storage. The effect on shelf‐life elongation was expected, as smoking is known to inactivate enzymes and microorganisms. However, smoking for 2.5 h did not have the same effect on extending the shelf life. After 2.5 h of smoking, the cheese samples exhibited microbial properties similar to fresh ones. In a study on Oscypek cheese, it was found that smoking did not affect TMAB counts at the beginning of shelf life, but reduced yeast and mold counts (Pyz‐ŁUkasik et al., 2018). In our study, according to the analysis of the first day of storage, both types of microorganisms were not affected by smoking. Scarano et al. (2019) also determined that 3 h smoking had no effect on yeast and mold counts in ricotta cheese, only the storage period reduced the microbial quality of the cheeses.

**TABLE 4:** The microbial counts of cheeses.

	Summer				Winter			
Storage day	1	30	60	90	1	30	60	90
TMAB (log.Cfu/g)								
70‐F	1.91 ± 0.19	2.74 ± 0.38	2.93 ± 0.80	3.87 ± 0.78	2.52 ± 0.26	4.75 ± 0.04	5.55 ± 0.30	5.77 ± 0.57
	a A	a A	a A	a A	a B	a A	a A	a A
70‐LS	1.44 ± 0.12	2.32 ± 0.60	2.71 ± 0.19	3.34 ± 0.41	2.67 ± 0.42	3.62 ± 0.97	4.69 ± 0.19	4.53 ± 0.68
	a B	a AB	a AB	ab A	a A	ab A	ab A	ab A
70‐HS	1.49 ± 0.10	1.90 ± 1.64	2.17 ± 0.29	2.67 ± 0.52	2.61 ± 1.00	2.45 ± 0.59	3.51 ± 1.17	4.04 ± 0.94
	a A	a A	ab A	abc A	a A	bc A	ab A	abc A
90‐F	1.19 ± 0.21	1.36 ± 0.55	1.80 ± 0.26	2.04 ± 0.07	1.42 ± 0.36	4.43 ± 0.28	5.10 ± 0.49	5.76 ± 0.40
	ab A	a A	ab A	bc A	a B	a A	a A	a A
90‐LS	1.19 ± 0.27	1.68 ± 0.91	1.73 ± 0.31	2.08 ± 0.14	1.45 ± 0.27	3.32 ± 0.40	3.65 ± 1.43	3.46 ± 0.53
	ab A	a A	ab A	bc A	a A	abc A	ab A	bc A
90‐HS	0.50 ± 0.50	0.78 ± 0.38	1.23 ± 0.20	1.39 ± 0.60	1.64 ± 0.07	1.54 ± 0.37	1.88 ± 0.27	2.14 ± 0.43
	b A	a A	b A	c A	a A	c A	b A	c A
Total yeasts and molds (log.Cfu/g)								
70‐F	0.29 ± 0.29	1.22 ± 0.20	1.94 ± 0.28	3.46 ± 0.29	0.33 ± 0.58	1.49 ± 0.37	2.38 ± 0.28	3.49 ± 0.31
	a C	a BC	a B	a A	a C	a BC	a AB	a A
70‐LS	0.62 ± 0.36	1.25 ± 0.58	1.99 ± 0.26	2.89 ± 0.09	0.33 ± 0.58	0.77 ± 0.35	0.81 ± 0.73	1.51 ± 0.41
	a C	a BC	a AB	a A	a A	a A	a A	a A
70‐HS	0.00 ± 0.00	0.00 ± 0.00	0.12 ± 0.21	1.40 ± 0.81	0.33 ± 0.58	0.67 ± 0.58	1.04 ± 0.06	1.23 ± 0.33
	a A	a A	b A	bc A	a A	a A	a A	a A
90‐F	0.24 ± 0.30	1.11 ± 0.19	1.50 ± 0.10	2.74 ± 0.31	0.00 ± 0.00	1.10 ± 0.18	2.18 ± 0.14	2.67 ± 0.31
	a C	a BC	ab B	ab A	a C	a B	a A	a A
90‐LS	0.50 ± 0.50	0.99 ± 0.99	1.29 ± 1.12	2.93 ± 0.23	0.00 ± 0.00	0.76 ± 0.67	1.55 ± 0.95	1.30 ± 1.33
	a A	a A	ab A	a A	a A	a A	a A	a A
90‐HS	0.00 ± 0.00	0.00 ± 0.00	0.00 ± 0.00	1.00 ± 0.00	0.00 ± 0.00	0.90 ± 0.18	1.80 ± 0.26	1.58 ± 0.52
	a	a	b	c	a B	a AB	a A	a A

*Note*: 70 and 90: *coagulation temperatures* (°C), ‐F: *fresh*, ‐LS: *2.5 h smoked*, ‐HS: *6 h smoked*, TMAB: *total mesophilic aerobic bacteria*. Lowercase letters in the same column indicate differences between samples on the same storage day (*p* < .01). Capital letters in the same line represent the change in the sample during the storage time. (*p* < .01).

### 3.3 Ripening properties

The ripening properties of cheeses were evaluated in terms of pH, acidity, WSN, RD, and TFFA. Results were given in Tables 5 and 6. Although the pH of the milk supplied was the same, it was observed that there were significant differences in the pH of the cheeses according to the season, while there was no significant difference in their acidity (Table 5). The higher pH of the winter cheeses may be related to its buffering capacity. Chen et al. (2014) determined that the pH of raw milk in winter is higher than that of summer milk, but the buffering capacity does not differ significantly throughout the year. Li et al. (2020), on the other hand, stated that pH and the degree of acid gel formation were associated with the lactation period, and low gelation rate and high buffering capacity were observed in the late lactation period. In our study, the effects of lactation time have not been studied. However, according to statistical evaluations, it can be concluded that the buffering capacity of winter milk used is higher than that of summer milk. For instance, while a significant pH decrease was observed in 90 days in fresh (F) and 2.5 h smoked (LS) cheeses produced with summer milk, the pH of cheeses produced with winter milk remained similar throughout the shelf life. Titration acidity also revealed a lower increase in winter milk compared to summer milk. On the other hand, it is known that smoke can lower the pH of foods due to the natural carboxylic acids in its composition (Rizzo et al., 2022). However, in our study, smoking up to 6 h did not affect the pH of the samples at the beginning of the shelf life. On the other hand, pH decrease was observed in all products during the storage, while a slower decline in long‐term smoked products. This trend was probably due to a combined effect: Microbial inhibition due to the drying effect of smoking, microbial and enzymatic inactivation due to volatile components of the smoke, and subsequently slowing of the rates of lipolysis and proteolysis. In this study, the phenolic contents of cheeses were not examined, but it was stated in the literature that smoke has a bactericidal effect as a result of the combination of its chemical components with drying (Ahmad, 2003).

**TABLE 5:** The pH and titratable acidity (%) of cheeses during 90 days of storage.

	Summer				Winter			
Storage day	1	30	60	90	1	30	60	90
pH								
70‐F	5.64 ± 0.03	5.54 ± 0.02	5.47 ± 0.00	5.41 ± 0.02	5.86 ± 0.14	5.87 ± 0.05	5.77 ± 0.25	5.57 ± 0.07
	bc A	c B	c C	b C	a A	a A	a A	a A
70‐LS	5.61 ± 0.01	5.55 ± 0.03	5.48 ± 0.01	5.44 ± 0.02	5.87 ± 0.11	5.87 ± 0.02	5.77 ± 0.18	5.71 ± 0.27
	c A	bc A	bc B	b B	a A	a A	a A	a A
70‐HS	5.59 ± 0.07	5.53 ± 0.04	5.49 ± 0.07	5.45 ± 0.03	5.87 ± 0.15	5.87 ± 0.01	5.81 ± 0.11	5.74 ± 0.26
	c A	c A	bc A	b A	a A	a A	a A	a A
90‐F	5.76 ± 0.01	5.64 ± 0.04	5.61 ± 0.04	5.60 ± 0.03	5.90 ± 0.06	5.90 ± 0.01	5.83 ± 0.08	5.71 ± 0.04
	a A	ab B	ab B	a B	a A	a A	a A	a A
90‐LS	5.73 ± 0.02	5.66 ± 0.01	5.64 ± 0.04	5.60 ± 0.03	5.90 ± 0.08	5.87 ± 0.05	5.84 ± 0.07	5.77 ± 0.06
	ab A	a AB	a AB	a B	a A	a A	a A	a A
90‐HS	5.70 ± 0.02	5.67 ± 0.01	5.67 ± 0.01	5.65 ± 0.04	5.92 ± 0.03	5.91 ± 0.02	5.89 ± 0.03	5.86 ± 0.09
	abc A	a A	a A	a A	a A	a A	a A	a A
Acidity (%LA)								
70‐F	0.44 ± 0.03	0.64 ± 0.04	0.82 ± 0.03	1.11 ± 0.01	0.42 ± 0.05	0.68 ± 0.07	0.78 ± 0.04	1.20 ± 0.06
	ab D	a C	a B	a A	a C	a B	a B	a A
70‐LS	0.47 ± 0.03	0.61 ± 0.08	0.74 ± 0.05	0.89 ± 0.06	0.47 ± 0.04	0.57 ± 0.05	0.71 ± 0.06	1.01 ± 0.06
	ab C	a BC	ab AB	bc A	a C	ab BC	a B	ab A
70‐HS	0.53 ± 0.00	0.62 ± 0.07	0.72 ± 0.01	0.88 ± 0.08	0.50 ± 0.05	0.55 ± 0.04	0.69 ± 0.07	0.95 ± 0.10
	a C	a BC	b B	c A	a B	abc B	a B	bc A
90‐F	0.42 ± 0.03	0.57 ± 0.06	0.76 ± 0.01	1.03 ± 0.03	0.38 ± 0.03	0.52 ± 0.02	0.64 ± 0.05	0.97 ± 0.07
	b D	a C	ab B	ab A	a C	bc B	a B	ab A
90‐LS	0.48 ± 0.05	0.55 ± 0.06	0.69 ± 0.05	0.87 ± 0.08	0.39 ± 0.06	0.47 ± 0.01	0.61 ± 0.03	0.85 ± 0.02
	ab C	a BC	b B	c A	a C	bc C	a B	bc A
90‐HS	0.47 ± 0.06	0.53 ± 0.02	0.67 ± 0.04	0.83 ± 0.05	0.41 ± 0.05	0.42 ± 0.01	0.56 ± 0.11	0.80 ± 0.05
	ab C	a C	b B	c A	a B	c B	a B	c A

*Note*: 70 and 90: coagulation temperatures (°C), ‐F: fresh, ‐LS: 2.5 h smoked, ‐HS: 6 h smoked. Lowercase letters in the same column indicate differences between samples on the same storage day (*p* < .01). Capital letters in the same line represent the change in the sample during the storage time. (*p* < .01).

**TABLE 6:** The proteolysis and lipolysis measurements of cheeses.

	Summer				Winter			
	1	30	60	90	1	30	60	90
WSN (%)								
70‐F	0.263 ± 0.015	0.294 ± 0.012	0.373 ± 0.017	0.433 ± 0.012	0.222 ± 0.011	0.335 ± 0.012	0.388 ± 0.007	0.487 ± 0.015
	a C	a C	a B	a A	a D	a C	a B	a A
70‐LS	0.285 ± 0.010	0.301 ± 0.010	0.364 ± 0.009	0.422 ± 0.009	0.232 ± 0.015	0.326 ± 0.020	0.381 ± 0.017	0.467 ± 0.019
	a C	a C	a B	a A	a C	a B	a B	a A
70‐HS	0.276 ± 0.016	0.292 ± 0.018	0.329 ± 0.017	0.414 ± 0.011	0.227 ± 0.011	0.301 ± 0.008	0.373 ± 0.008	0.445 ± 0.004
	a B	a B	a B	a A	a D	a C	a B	a A
90‐F	0.250 ± 0.017	0.327 ± 0.008	0.374 ± 0.010	0.427 ± 0.009	0.214 ± 0.006	0.322 ± 0.017	0.383 ± 0.011	0.485 ± 0.017
	a D	a C	a B	a A	a D	a C	a B	a A
90‐LS	0.272 ± 0.016	0.319 ± 0.013	0.337 ± 0.012	0.404 ± 0.010	0.233 ± 0.011	0.337 ± 0.010	0.384 ± 0.011	0.476 ± 0.014
	a C	a B	a B	a A	a D	a C	a B	a A
90‐HS	0.274 ± 0.019	0.304 ± 0.026	0.335 ± 0.024	0.386 ± 0.034	0.216 ± 0.020	0.296 ± 0.007	0.385 ± 0.019	0.448 ± 0.011
	a B	a AB	a AB	a A	a D	a C	a B	a A
RD (%)								
70‐F	9.77 ± 0.57	10.92 ± 0.45	13.82 ± 0.64	16.07 ± 0.44	7.83 ± 0.38	11.81 ± 0.43	13.69 ± 0.23	17.17 ± 0.52
	a C	a C	a B	a A	ab D	a C	a B	a A
70‐LS	10.32 ± 0.35	10.90 ± 0.35	13.19 ± 0.31	15.30 ± 0.31	8.08 ± 0.51	11.37 ± 0.71	13.27 ± 0.59	16.29 ± 0.65
	a C	a C	ab B	ab A	a C	a B	ab B	ab A
70‐HS	9.27 ± 0.54	9.79 ± 0.61	11.04 ± 0.58	13.90 ± 0.37	7.31 ± 0.34	9.71 ± 0.27	12.01 ± 0.27	14.35 ± 0.12
	a B	a B	c B	bc A	ab D	bc C	bc B	c A
90‐F	8.72 ± 0.59	11.40 ± 0.28	13.03 ± 0.36	14.89 ± 0.32	7.25 ± 0.22	10.93 ± 0.58	13.00 ± 0.37	16.46 ± 0.56
	a D	a C	ab B	ab A	ab D	ab C	abc B	ab A
90‐LS	9.12 ± 0.52	10.71 ± 0.44	11.32 ± 0.40	13.56 ± 0.33	7.41 ± 0.34	10.69 ± 0.30	12.19 ± 0.33	15.12 ± 0.44
	a C	a B	bc B	bc A	ab D	ab C	bc B	bc A
90‐HS	8.79 ± 0.61	9.75 ± 0.84	10.73 ± 0.76	12.38 ± 1.09	6.55 ± 0.60	8.99 ± 0.21	11.69 ± 0.58	13.62 ± 0.33
	a B	a AB	c AB	c A	b D	c C	c B	c A
TFFA (g/100 g fat)								
70‐F	0.102 ± 0.025	0.176 ± 0.023	0.199 ± 0.043	0.389 ± 0.022	0.131 ± 0.042	0.211 ± 0.026	0.224 ± 0.015	0.303 ± 0.024
	a B	b B	b B	c A	a B	b AB	b AB	d A
70‐LS	0.131 ± 0.016	0.225 ± 0.025	0.264 ± 0.031	0.483 ± 0.009	0.129 ± 0.029	0.214 ± 0.048	0.241 ± 0.023	0.347 ± 0.016
	a C	b B	b B	b A	a C	b BC	b AB	cd A
70‐HS	0.184 ± 0.038	0.366 ± 0.001	0.458 ± 0.029	0.737 ± 0.016	0.191 ± 0.040	0.351 ± 0.013	0.465 ± 0.029	0.672 ± 0.019
	a D	a C	a B	a A	a D	a C	a B	a A
90‐F	0.099 ± 0.046	0.192 ± 0.035	0.226 ± 0.007	0.416 ± 0.012	0.135 ± 0.048	0.191 ± 0.023	0.254 ± 0.013	0.411 ± 0.044
	a C	b BC	b B	c A	a B	b B	b B	b A
90‐LS	0.122 ± 0.050	0.225 ± 0.017	0.273 ± 0.019	0.491 ± 0.022	0.146 ± 0.029	0.230 ± 0.049	0.317 ± 0.037	0.498 ± 0.022
	a C	b BC	b B	b A	a C	b BC	b B	bc A
90‐HS	0.155 ± 0.011	0.387 ± 0.022	0.468 ± 0.024	0.716 ± 0.014	0.174 ± 0.036	0.395 ± 0.031	0.501 ± 0.044	0.758 ± 0.025
	a D	a C	a B	a A	a C	a B	a B	a A

*Note*: 70 and 90: *coagulation temperatures* (°C), ‐F: *fresh*, ‐LS: *2.5 h smoked*, ‐HS: *6 h smoked*, WSN: *water‐soluble nitrogen*, RD: *ripening degree*, TFFA: *total free fatty acids*. Lowercase letters in the same column indicate differences between samples on the same storage day (*p* < .01). Capital letters in the same line represent the change in the sample during the storage time. (*p* < .01).

When the results in Table 6 were evaluated, it was determined that the seasonal change in milk had no effect on the proteolysis and lipolysis rates of cheeses. Smoking had no additional effect on the WSN ratio, and a significant increase in proteolysis was observed in all products on the 90th day of storage. Shakeel‐Ur‐Rehman et al. (2003) also determined that WSN ratios in Cheddar cheeses were not affected by the cold‐smoking process. The increase in RD accelerated with increasing smoking time.

No difference was observed in the TFFA values at the beginning of the shelf life of the cheeses. However, while TFFA increased in all products during the storage, the rate of increase progressed proportionally with the increase in smoking time. This can be explained by the enzyme inactivation property of the smoking process. The compounds that give the smoke flavor are usually phenols, carbonyl compounds, and organic acids. They reduce microbiological contamination and inhibit lipid oxidation as a result of its antioxidant effect (Dopieralska et al., 2020). According to the results, lipid oxidation was inhibited more with increasing smoking time. Similar results were obtained in a study examining the smoking and storage process of Idiazabal cheese (Nájera et al., 1994). In a market study of smoked cheeses, it was determined that smoking increases the concentration of short‐chain fatty acids rather than saturated ones (Paszczyk et al., 2020). In the shelf‐life study of Domiati cheese, as in our study, the amount of TFFA increased 3–4 times during the 90‐day storage period (Ammar et al., 2015).

### 3.4 Principle component analysis of ripening properties

**FIGURE 1:**
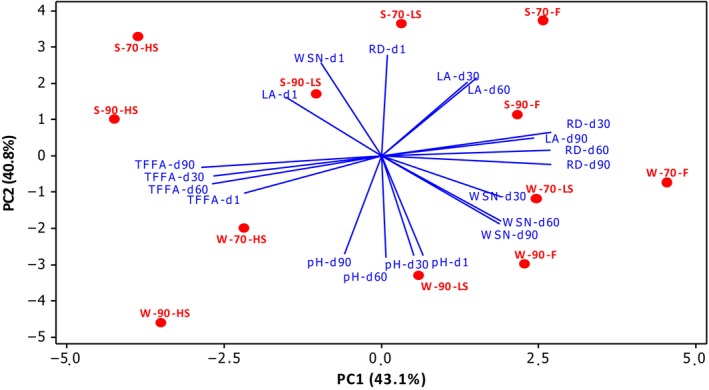
Biplot of the first two principal components of ripening properties of cheeses on the storage days 1, 30, 60, and 90. WSN, RD, TFFA, pH, and LA represent the types of ripening properties. The S and W abbreviations represent the milking seasons of summer and winter, respectively, and the following numbers (70 or 90) represent the coagulation temperature (°C). The F, LS, and HS abbreviations following the temperature represent groups of cheeses that have been smoked at different times.

Principal component analysis was applied to clarify the effects of storage time, smoking process, coagulation temperature differences, and seasonal variation on the ripening properties of cheeses, and the results are given in Figure 1. The two main components explaining 83.9% of the total variance are given as PC1 (43.1%) and PC2 (40.8%) in Figure 1, respectively. Seasonal variation in milk significantly affected the ripening properties of cheeses. Cheeses produced with summer milk were on the positive side of PC2, and those produced with winter milk were on the negative side of PC2. Another remarkable group was also obtained according to PC1. While fresh cheeses (F groups) were on the positive side of PC1, they progressed to the negative area by smoking. The change in coagulation temperature was the parameter that least affected the ripening properties of the products. WSN values were in the negative area of PC1 and positive area of PC2 on the first day of storage, while as the storage progressed, they were located in the opposite direction. On the other hand, TFFA progressed in the opposite direction of RD. The lactic acid (LA) and pH were also located in opposite directions, as expected. The storage time also affected the pH and LA results. The LA, which was located in the positive direction of PC2, progressed from the negative area of PC1 to the positive during storage. The pH was located in the negative direction of PC2 and moved from the positive area of PC1 to the negative during storage.

### 3.5 Textural evaluation

The hardness values of the cheeses are given in Table 7. It has been observed that seasonal changes in milk caused a significant textural difference in most of the products. The higher hardness values of winter cheeses were probably due to their higher DM contents. Similarly, cheeses produced by coagulating at 70°C had lower hardness values, as expected, since they had lower DM content than products coagulated at 90°C. In the study of Hydamaka et al. (2001), as in our results, milk that was heated to 90°C and coagulated after cooling to 70°C formed a softer cheese than that coagulated at 90°C.

**TABLE 7:** The hardness values of cheeses during 90 days of storage.

	Hardness (g)							
	Summer				Winter			
Storage day	1	30	60	90	1	30	60	90
70‐F	890 ± 50	571 ± 62	608 ± 75	520 ± 53	855 ± 33	721 ± 48	807 ± 80	707 ± 82
	c A	c B	d B	d B	d A	d A	c A	d A
70‐LS	1011 ± 86	694 ± 112	854 ± 12	834 ± 55	1061 ± 60	861 ± 56	853 ± 57	841 ± 49
	c A	c B	d AB	c AB	c A	d B	c B	cd B
70‐HS	1131 ± 94	967 ± 31	1169 ± 64	1056 ± 127	1086 ± 64	1099 ± 76	914 ± 87	954 ± 33
	bc A	b A	c A	c A	c A	c A	c A	c A
90‐F	1287 ± 76	1127 ± 72	1503 ± 109	1516 ± 82	1496 ± 61	1652 ± 29	1382 ± 11	1332 ± 81
	b AB	b B	b A	b A	b AB	b A	b B	b B
90‐LS	1327 ± 62	1072 ± 66	1596 ± 58	1697 ± 91	1631 ± 27	1731 ± 76	1696 ± 22	1408 ± 68
	b B	b C	b A	b A	b A	b A	a A	b B
90‐HS	1774 ± 61	1603 ± 42	2015 ± 79	2055 ± 52	2017 ± 58	1986 ± 88	1876 ± 10	1891 ± 80
	a B	a B	a A	a A	a A	a A	a A	a A

*Note*: 70 and 90: *coagulation temperatures* (°C), ‐F: *fresh*, ‐LS: *2.5 h smoked*, ‐HS: *6 h smoked*. Lowercase letters in the same column indicate differences between samples on the same storage day (*p* < .01). Capital letters in the same line represent the change in the sample during the storage time. (*p* < .01).

In both summer and winter cheeses, it was determined that 6 h of smoking caused a significant increase in cheese hardness during storage, while it was observed that 2.5 h of smoking caused a hardness value closer to the fresh product. It is thought that the DM differences between the products are effective in obtaining these results. Tunick et al. (2008), on the other hand, reported contrary to our results that, there was no significant change in the texture profile and microstructure of queso blanco cheese after approximately 90 days of storage.

### 3.6 Sensorial properties

**FIGURE 2:**
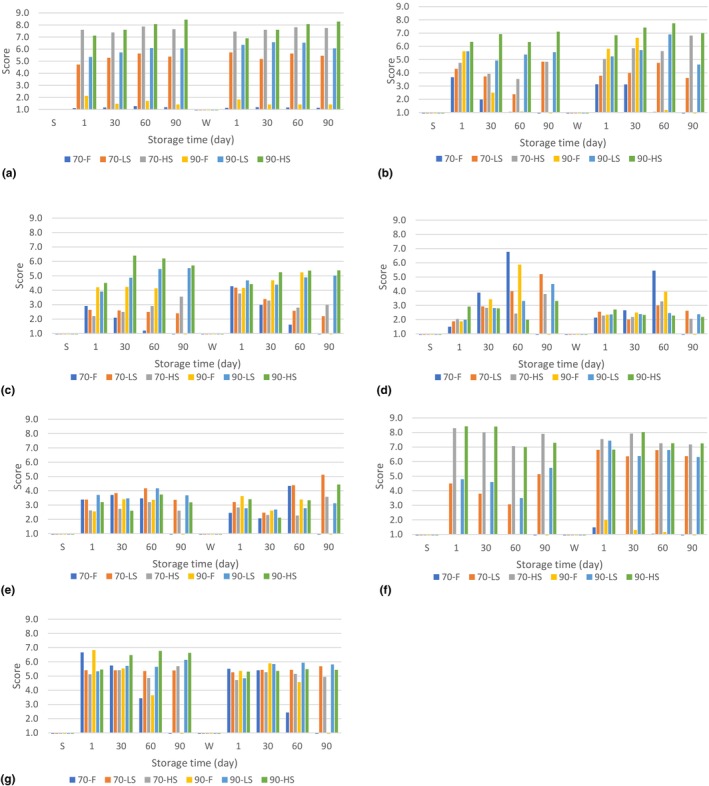
Sensorial evaluations of cheeses. (a) Color, (b) hardness, (c) elasticity, (d) sourness, (e) saltiness, (f) smoked flavor intensity, (g) general acceptance. S: *summer*, W: *winter*, 70 and 90: *coagulation temperatures* (°C), ‐F: *fresh*, ‐LS: *2.5 h smoked*, ‐HS: *6 h smoked*

Sensory analysis results are given in Figure 2. Fresh cheeses were not evaluated on the 90th day of storage except for color evaluation, to avoid possible microbial risk.

As a result of the sensory evaluation, it was determined that the cheese colors were not affected by seasonal changes. It was also determined that the 2.5 h and 6 h smoking processes caused a significant increase in yellowness in color (*p* > .01). According to the literature, the smoking time (Škaljac et al., 2018) as well as the smoking temperature varying between 20 and 30°C (Cardinal et al., 2001) has a determining effect on the color intensity.

The hardness of the summer cheeses was generally lower than the winter cheeses. When the effect of smoking was examined, it was determined that 6 h of smoking significantly contributed to the increase in hardness of the products and the preservation of this texture throughout the shelf life. Softening was observed during the shelf life of unsmoked fresh products. Excessive proteolysis results in a bitter taste and short‐textured cheese (Fröhlich‐Wyder & Bachmann, 2004). The parameters affecting the texture of the cheese are microscale and complex systems. The hardness scores on the sensory analysis support the analytical measurement: less smoked cheeses were generally scored similar to fresh cheeses, while 6‐h smoked products were scored as significantly harder, and products coagulated at 70°C were found to be softer than those coagulated at 90°C.

The number of chains of the calcium phosphate *para*‐casein network per unit area of cheese is a measure that determines gel strength and elasticity (Guinee, 2016). In addition, the disulfide bonds between casein and β‐lactoglobulin in acid‐heat coagulated cheeses contribute significantly to gel formation and elasticity (Fox et al., 2017). Both the pH of the product and the type of acid used to make the cheese curd play important roles in the level of elasticity (Bansal & Veena, 2022; Seth & Bajwa, 2015). It has also been reported that the type of wood used in smoking has a significant effect on the elasticity of the product (Fresno et al., 2005). Due to the complex structure of cheeses, there are many parameters that affect elasticity as well as other textural properties. In the sensory analysis results of our study, it was determined that seasonal change did not affect the elasticity of cheeses. Products generally remained the same in elasticity throughout their shelf life, and cheeses coagulated at 90°C were found to be more elastic than those coagulated at 70°C. When the results of first day analyses were evaluated, it was observed that the smoking process did not have a significant effect on the elasticity of the product (*p* > .01). However, in the other analyses days, some of the smoked products scored higher in elasticity than fresh products, possibly due to differences in ripening levels during the shelf life.

The effect of seasonal changes on cheese sourness was not significant at the beginning of its shelf life. However, winter cheeses were found to be sourer at the end of storage. Sourness progressed rapidly during storage in fresh products (70‐F and 90‐F), but no significant change was observed in 6 h smoked (70‐HS and 90‐HS) samples. This is thought to be due to the difference in ripening rates of fresh and smoked products. In addition to the lactic acid added during production, the acids formed by the proteolysis of cheese contribute to the sourness of the cheese (Kilcawley, 2017). The change in coagulation temperature did not affect the sourness of the products at the beginning of the shelf life, but on the 60th day, the products coagulated at 90°C were found to be less sour than those coagulated at 70°C.

It is stated in the literature that changes in product formulation and fat content cause perception differences in smoked flavor and salty taste intensity (Shamil et al., 1991). Our study revealed that both the 2.5 and 6 h smoking processes do not have a significant effect on the salty taste perception. Kostyra and Baryłko‐Pikielna (2007) also showed that smoking did not change the perception of salty taste, as in our study. In addition, coagulation temperature and storage time did not affect the saltiness of the cheeses in our study. Panelists rated all products as “low salted.” In further studies, the salt level should be increased within the limits allowed by the regulation and the salt content should be determined after a panel test of at least 100 people.

The aromatic composition of smoke obtained by the pyrolysis of wood varies according to the source of the tree from which it is obtained (Fresno et al., 2005). In addition, it has been stated in the literature that the smoked flavor can be detected by the panelists even in fresh cheeses (Giaccone et al., 2016). In our study, the smoked flavor was also detected in fresh cheeses. The smoke flavor intensity of cheeses smoked for 2.5 h was evaluated as “optimal,” and that of the cheeses smoked for 6 h as “extremely high.” However, since the flavor intensity is also a liking test, it will be necessary to expand the panel group in future studies.

Although the overall acceptability scores of fresh products were higher on the first day of storage, the scores of these products decreased as the shelf life progressed. On the 60th storage day, fresh cheeses were not liked, while smoked cheeses scored over 5 points. The positive effect of the smoking process on storage stability was clearly observed in this type of evaluation.

### 3.7 Principle component analysis of sensory properties

**FIGURE 3:**
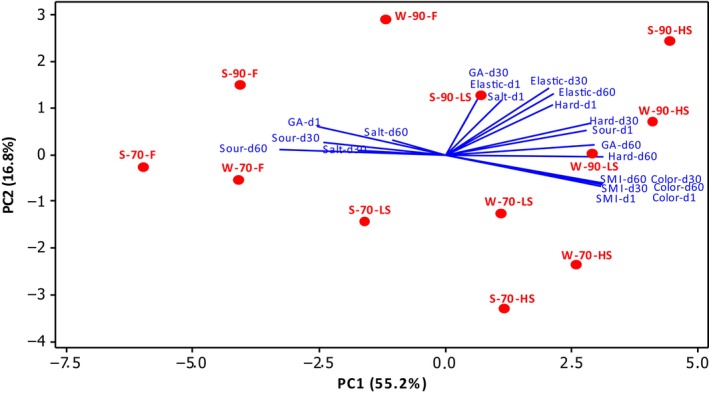
Biplot of the first two principal components of sensory properties of cheeses on the storage days 1, 30, and 60. The abbreviations of the types of sensory properties are as follows: Sour – sourness, salt – saltiness, elastic – elasticity, hard – hardness, color – color, SMI – smoked flavor intensity, and GA – general acceptance. The S and W abbreviations represent the milking seasons of summer and winter, respectively, and the following numbers (70 or 90) represent the coagulation temperature (°C). The F, LS, and HS abbreviations following the temperature represent groups of cheeses that have been smoked at different times.

The evaluation of sensory properties by using principal component analysis was made on days 1, 30, and 60 of storage, as fresh products could not be analyzed on day 90. The plot is shown in Figure 3. According to PC2, the products can be classified by sourness, saltiness, elasticity, hardness, and general acceptance in one group, and by color and smoked flavor intensity in the other group. According to PC1, sourness and saltiness formed one group on the 30th and 60th storage days with the analysis of general acceptance on the first day, while all other parameters were in the other group. On the 30th and 60th days of storage, the sourness and saltiness properties of fresh products were dominant, and these properties decreased as the smoking time increased. The application of smoking for 6 h increased the hardness and elasticity properties of both summer and winter cheeses. The texture of fresh products was found to be weaker than the smoked ones. The application of coagulation at 90°C also provided a harder and more elastic cheese than at 70°C. As the smoking time increased, the color scores and intensity of smoked flavor increased, and sourness and saltiness were perceived to be lower in these products. The cheeses produced with winter milk were found to be less sour, harder, and more elastic than the summer cheeses, in most of the samples. On the first day of storage, fresh products received high scores in terms of general acceptance, while 6‐h smoked (HS) products were more appreciated on the 60th day of storage.

### 3.8 Microstructures of the cheeses

**FIGURE 4:**
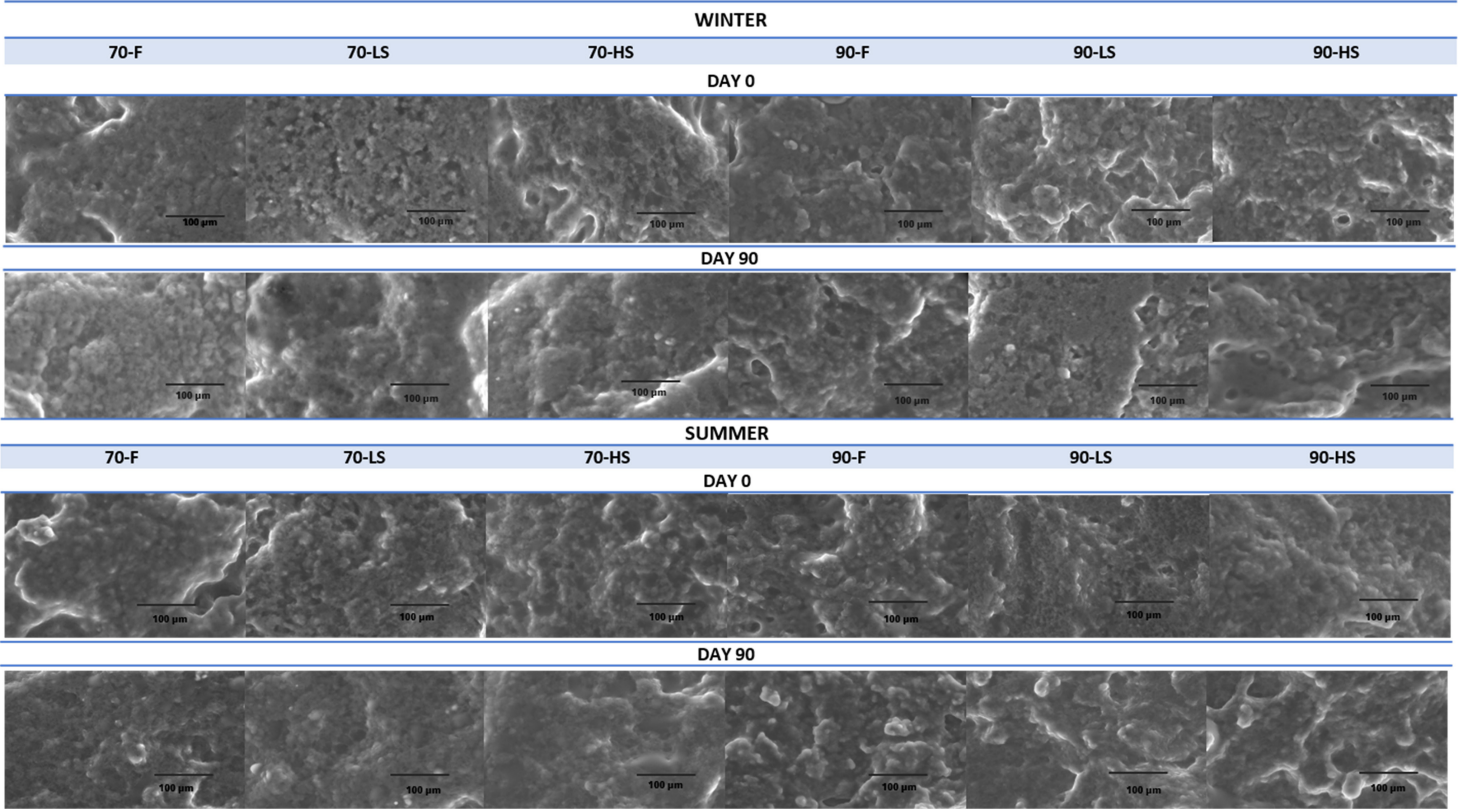
Microstructures of cheeses on day 0 and 90 (70 and 90: coagulation temperatures [°C]), ‐F: *fresh*, ‐LS: *2.5 h smoked*, ‐HS: *6 h smoked*.

The microstructure of cheese is a complex system in the form of a protein matrix that includes many components such as fat, water, minerals, bacterial colonies, and metabolites. This structure is a living system that is constantly changing during the production, ripening, and storage of cheese (Lucey et al., 2003). Its physicochemical properties and nutritional bioavailability are also related to microstructure (Parada & Aguilera, 2007). Macro properties such as hardness and elasticity are controlled by the microstructure. So, it is important to know the microstructure of the cheese in order to obtain the desired texture. The microstructures of the cheeses produced in this study are given in Figure 4. In the images obtained with 1000x magnification, globular fat and combined fat masses dispersed in the protein matrix and hollow structures were observed. When the microstructures of fresh and smoked types of cheeses were examined on the 1st and 90th storage days, it was observed that the protein matrix was more uniform and more continuous, and protein clusters were smaller in cheeses coagulated at 70°C. In cheeses coagulated at 90°C, hollows were bigger, and a coarse and irregular structure was observed. It was reported that the hydrophobic interactions that enhance the aggregation of casein particles are more common at high temperatures (Madadlou et al., 2006), and the deformation of the protein network occurs with the increase of casein aggregate formation (Lucey et al., 1997). When the cheeses were coagulated at 90°C, the dense of casein particle aggregation resulted in new protein strand formation and an increase in endogenous pressure that promotes syneresis (van Vliet et al., 1991). These rearrangements caused more aggregations and larger hollows in the cheeses coagulated at 90°C when compared to those coagulated at 70°C. It has been observed that increases in smoking time also supported syneresis and increased hollow diameters. On the 90th day of storage, individual fat globules were replaced by aggregated fat masses, probably due to the breakdown of the casein network, which holds the fat globules in place, as also reported by Tunick et al. (1993) and Fox et al. (2017). In addition, no microstructural differences were observed between summer and winter cheeses.

## 4 CONCLUSION

Seasonal changes, coagulation temperature, and smoking time affected the dry matter of the products. Higher dry matter and firmer texture was provided by 90°C coagulation and 6‐h smoking process. Higher smoking times (6‐h smoking) also reduced microbial growth, sourness, and proteolysis during the storage of cheeses. However, flavor was intense and lipolysis accelerated with 6 h of smoking.

In conclusion, the data obtained in this study will be a guide for standardizing the Circassian cheese production process. A smoking time of 2.5–6 h can be applied to ensure sensory attributes as well as improve other quality characteristics at the same time. Further research is needed to identify the Circassian cheese prior to the PDO registration. For instance, products produced through a standardized process should be characterized by volatile and microbiota fingerprints.

## AUTHOR CONTRIBUTIONS

**Hatice Sıçramaz:** Conceptualization (equal); data curation (equal); formal analysis (lead); investigation (equal); methodology (equal); writing – original draft (lead); writing – review and editing (equal). **Ahmet Ayar:** Conceptualization (equal); data curation (equal); funding acquisition (lead); investigation (equal); methodology (equal); project administration (lead); writing – original draft (supporting); writing – review and editing (equal).

## CONFLICT OF INTEREST STATEMENT

The authors declare no conflict of interest.

## Data Availability

Data available on request due to privacy/ethical restrictions.
